# Application of Radionuclide Myocardial Imaging in the Diagnosis and Treatment of Heart Failure With Preserved Ejection Fraction

**DOI:** 10.31083/RCM41231

**Published:** 2025-10-27

**Authors:** Yu Tian, Yuetao Wang, Jianfeng Wang, Xiaoliang Shao, Feifei Zhang

**Affiliations:** ^1^Department of Nuclear Medicine, The Third Affiliated Hospital of Soochow University, 213003 Changzhou, Jiangsu, China; ^2^Institute of Clinical Translation of Nuclear Medicine and Molecular Imaging, Soochow University, 213003 Changzhou, Jiangsu, China

**Keywords:** heart failure with preserved ejection fraction, radionuclide imaging, positron emission tomography, single-photon emission computed tomography, molecular imaging

## Abstract

Heart failure with preserved ejection fraction (HFpEF) represents a major phenotype of heart failure and accounts for over 50% of clinical cases. The complex pathophysiological mechanism involved in HFpEF promotes diagnostic difficulties and limited treatment options, posing a significant challenge in modern cardiology. Conventional imaging methods have significant limitations in comprehensively evaluating the heterogeneous etiologies and key pathological mechanisms of HFpEF. Radionuclide myocardial imaging, through the application of targeted radioactive tracers, enables *in vivo*, non-invasive quantitative assessment of multiple pathological and physiological processes such as myocardial perfusion, energy metabolism, sympathetic nervous activity, inflammatory responses, and fibrotic progression. Moreover, this technology offers a transformative approach to the precise diagnosis, molecular phenotyping, risk stratification, therapeutic monitoring, and prognostic assessment of HFpEF. Therefore, this review systematically summarizes the latest progress in radionuclide myocardial imaging techniques in diagnosing and treating HFpEF, with a particular focus on analyzing the unique clinical value of this technology in identifying specific etiologies (such as cardiac amyloidosis, cardiac sarcoidosis, and coronary microvascular dysfunction) and elucidating pathological mechanisms (including metabolic remodeling, inflammatory, fibrosis, and alterations in sympathetic innervation). Furthermore, we discuss the future directions of this imaging modality, including the development of novel molecular probes, integration with multimodal imaging techniques, and the application of artificial intelligence-assisted analysis. These innovations are expected to facilitate a paradigm shift from symptom-oriented management to mechanism-targeted therapy, offering new perspectives for the precise classification and clinical management of HFpEF.

## 1. Introduction

Heart failure with preserved ejection fraction (HFpEF) accounts for more than 50% of heart failure cases. Its incidence has been continuously rising along with an aging population and the growing prevalence of metabolic diseases such as diabetes and obesity [1, 2]. Unlike heart failure with reduced ejection fraction (HFrEF), HFpEF is characterized by preserved left ventricular ejection fraction (LVEF) ≥50% in the presence of distinct pathophysiological abnormalities, including diastolic dysfunction, elevated pulmonary artery pressures, abnormal myocardial energy metabolism, microcirculation dysfunction and myocardial fibrosis. At present, the clinical diagnosis and treatment of HFpEF remain challenging, primarily due to its substantial heterogeneity and complex, multifactorial etiology. Although the diagnosis of HFpEF primarily relies on clinical symptoms, echocardiography and biomarkers such as N-terminal pro-B-type natriuretic peptide (NT-proBNP); the lack of specific diagnostic markers poses significant challenges for early detection and precise phenotyping [1, 3, 4]. In addition to conventional risk factor control and the use of novel agents such as sodium-glucose cotransporter 2 (SGLT2) inhibitors, targeted therapies for specific etiologies (e.g., transthyretin cardiac amyloidosis) are constantly evolving and improving [1]. Approximately 10 to 15% of patients clinically diagnosed with HFpEF may actually have conditions that mimic heart failure symptoms but possess distinct etiologies and treatment strategies, such as cardiac amyloidosis or sarcoidosis. Accurate identification of these ‘masquerade syndromes’ is crucial for guiding appropriate therapy and improving patient outcomes [5, 6].

Cardiovascular imaging techniques are crucial in the diagnosis and etiology of HFpEF. Echocardiography and cardiac magnetic resonance (CMR) are widely utilized imaging modalities for assessing structural and functional abnormalities, including left ventricular diastolic dysfunction and myocardial fibrosis. However, conventional imaging modalities are limited in their ability to assess key mechanisms such as microvascular dysfunction, myocardial metabolic abnormalities, and sympathetic dysregulation, making it challenging to fully reveal the underlying causes and key pathophysiological mechanisms of HFpEF. Radionuclide myocardial imaging, a functional molecular imaging technique, enables multidimensional evaluation of myocardial perfusion, metabolism, sympathetic nerve activity, inflammation, and fibrosis through radioactive tracer technology. It not only helps to identify specific etiologies (e.g., cardiac amyloidosis, sarcoidosis, coronary microvascular dysfunction), but also can deeply analyze key pathophysiological mechanisms such as myocardial metabolic abnormalities, inflammation, sympathetic nerve imbalance, and fibrosis [7]. This provides a unique perspective for the precise phenotyping and individualized management of HFpEF.

This review aims to comprehensively explore the clinical application of radionuclide myocardial imaging in HFpEF, with a focus on its unique value in identifying specific etiologies such as cardiac amyloidosis, cardiac sarcoidosis, and coronary microvascular disease and elucidating pathophysiological mechanisms including inflammation, fibrosis, metabolic remodeling, sympathetic nerve function, and cardiac function. It also offers novel insights for the precise diagnosis of HFpEF, optimization of clinical management, improvement of patient prognosis, and future research directions, thereby promoting the shift from traditional symptom-based management to mechanism-targeted therapeutic strategies.

## 2. Literature Review

### 2.1 Radionuclide Myocardial Imaging

Radionuclide myocardial imaging mainly relies on positron emission tomography (PET) and single photon emission computed tomography (SPECT) [7]. Due to its broad availability and relatively low cost, SPECT is widely utilized in routine clinical practice for evaluating myocardial perfusion, sympathetic nerve activity, and cardiac amyloidosis. PET, with its outstanding spatial resolution and precise quantitative analysis capabilities, is particularly adept at accurately assessing myocardial metabolic status, microvascular function, and inflammatory responses. It is valuable in the diagnosis, classification, mechanistic analysis, prognosis evaluation, and treatment monitoring of HFpEF.

In clinical practice, various radioactive tracers provide powerful tools for the assessment of different pathophysiological processes. The applicable scope, usage precautions and limitations of common tracers are detailed in Table 1 (Ref. [8, 9, 10, 11, 12, 13]). In the evaluation of myocardial perfusion, commonly used tracers include ^99m^technetium-methoxy isobutyl isonitrile (^99m^Tc-MIBI) and ^201^thallium (^201^Tl) for SPECT, as well as ^13^N-ammonia, ^15^O-water and ^82^rubidium (^82^Rb) for PET. The latter allows for quantitative measurement of myocardial blood flow (MBF) and myocardial blood flow reserve (MFR). PET is considered the gold standard for noninvasively assessing regional MBF—and is especially valuable in detecting coronary microvascular dysfunction (CMD) in HFpEF patients. In metabolic imaging, ^18^F-fluorodeoxyglucose (^18^F-FDG) and Iodine-123 β-methyl iodophenyl pentadecanoic acid (^123^I-BMIPP) are used to detect abnormal glucose and fatty acid metabolism in the myocardium. In the diagnosis of specific etiologies, ^99m^Tc-pyrophosphate (^99m^Tc-PYP) imaging has significant diagnostic value for transthyretin cardiac amyloidosis (ATTR-CA). Recent advancements in molecular probes have opened new avenues for studying HFpEF. Tracers such as ^68^Gallium-labeled DOTA-(Tyr3)-Octreotate (^68^Ga-DOTATATE) and pentixafor can achieve precise imaging of myocardial inflammation [8]. Radiotracers targeting fibroblast activation protein (FAP), such as ^68^Gallium fibroblast activation protein inhibitor (^68^Ga-FAPI), have shown promising application in the early detection and quantitative assessment of myocardial fibrosis, providing new molecular imaging tools for the study of the pathological mechanism and individualized treatment of HFpEF. These technological advancements are advancing radionuclide imaging from traditional functional assessment towards precise molecular diagnosis. The indications, advantages and disadvantages of PET, SPECT, CMR and echocardiography are detailed in Table 2 (Ref. [1, 5, 7, 14, 15, 16]).

**Table 1.** **Applications of radiotracers in myocardial imaging for HFpEF**.

Radiotracer	Clinical application	Radiation dose	Notes for attention	Limitations
^18^F-FDG [11]	Evaluation of inflammation;	3.8–7.2 mSv	Maintain strict glycemic control (target ≤7.8 mmol/L);	Low specificity for etiology;
	Cardiac sarcoidosis diagnosis;		Fast for 6–12 hours pre-procedure; avoid glucose-containing IV fluids;	Susceptible to blood sugar fluctuations
	Ischemic memory;		Pre-treat with high-fat/low-carb diet;	
	Myocardial metabolic evaluation		Contraindications during pregnancy	
^99m^Tc-MIBI [12]	SPECT-based perfusion imaging;	7–9 mSv	Safe and widely used;	Semi-quantitative;
	Detection of CMD;		good availability	Limited MBF assessment
	Ischemia and Infarction			
^99m^Tc-PYP [13]	Diagnosis of ATTR-CA	∼10 mSv	Image acquisition 3 hours post-injection;	Not suitable for AL-CA;
			Perform semi-quantitative analysis with blood pool imaging;	Requires serum exclusion
			Dose adjustment required for severe renal impairment patients	
^123^I-MIBG [10]	Cardiac Sympathetic Function Assessment;	2.5–3.5 mSv	Discontinue tricyclic antidepressants 6 weeks prior;	Influenced by comorbidities (diabetes, CKD);
	Neuroendocrine Tumors		Thyroid blockade (potassium iodide 120 mg daily × 7 days);	Inter-individual variability
			Monitor heart rate variability post-injection;	
			Avoid use in acute phase of acute coronary syndrome	
^68^Ga-FAPI [9]	Imaging myocardial fibrosis and remodeling	3–5 mSv	Low background uptake;	Lacks large-scale validation
			Good image contrast	
^68^Ga-DOTATATE [8]	Myocardial Metastasis of Neuroendocrine Tumors;	4.5–5.5 mSv	Discontinue somatostat­in analogues 24–72 hours prior;	Clinical use in HFpEF is still exploratory;
	Targeted Molecular Imaging of M1 Macrophage-Associated Inflammation		Evaluate renal function (use with caution if GFR <30);	Quantitative analysis complexity;
			Thyroid blockade requires potassium iodide pre-treatment	Limited to certain inflammatory types

HFpEF, heart failure with preserved ejection fraction; ^18^F-FDG, ^18^Fluorine fluorodeoxyglucose; IV, intravenous; ^99m^Tc-MIBI, ^99m^technetium methoxy isobutyl isonitrile; SPECT, single-photon emission computed tomography; CMD, coronary microvascular dysfunction; MBF, myocardial blood flow; ^99m^Tc-PYP, ^99m^technetium pyrophosphate; ATTR-CA, transthyretin cardiac amyloidosis; AL-CA, light-chain (AL) cardiac amyloidosis; ^123^I-MIBG, ^123^I-metaiodobenzylguanidine; CKD, chronic kidney disease; ^68^Ga-FAPI, ^68^Gallium fibroblast activation protein inhibitor; ^68^Ga-DOTATATE, ^68^Gallium-labeled DOTA-(Tyr3)-Octreotate; GFR, glomerular filtration rate.

**Table 2.** **Comparison of imaging modalities in diagnosing etiology and pathophysiological mechanisms of HFpEF**.

Imaging modality	Targeted etiologies/mechanisms	Advantages	Limitations	Cost & availability
Echocardiography [1, 15]	Structural assessment;	Economical;	Limited tissue characterization;	Low cost;
	Wall motion-diastolic dysfunction;	Real-time, bedside;	Operator-dependent;	Widely available
	Amyloidosis (strain pattern)	Global and regional function;	Cannot directly detect inflammation/fibrosis	
		No radiation;		
		Safe for all populations		
CMR [14, 16]	Myocardial fibrosis;	High spatial resolution;	Contraindicated in some patients (devices);	Moderate cost;
	Infiltrative disease (amyloid/sarcoid);	Tissue characterization (LGE, T1/T2/ECV);	Contraindicated in severe renal dysfunction;	Moderate availability
	Edema/inflammation- Ischemia/perfusion;	Quantitative perfusion;	Long scan time;	
	CMD (perfusion reserve)	No ionizing radiation	Poor availability in some regions	
SPECT [5, 7]	Myocardial perfusion (ischemia/CAD);	High specificity;	Limited spatial resolution;	Moderate cost;
	ATTR amyloidosis (^99m^Tc-PYP/DPD);	Capable of prognostic evaluation;	Semi-quantitative;	Very accessible
	Sympathetic innervation (^123^I-MIBG);	Widespread clinical use	Radiation exposure;	
	Inflammation (^99m^Tc-HMPAO)		Less sensitive than PET for flow/metabolism;	
			Moderate radiation (5–10 mSv);	
			Avoid in pregnancy;	
			Caution in thyroid disease (MIBG)	
PET [7, 14]	Myocardial perfusion & MBF/MFR (CAD, CMD);	Highest quantitative accuracy;	Moderate radiation (4–10 mSv);	High cost;
	Inflammation (^18^F-FDG, ^68^Ga-DOTATATE);	Gold standard for CMD;	Contraindicated in pregnancy;	Limited in many centers
	Amyloidosis (^18^F-florbetapir, ^11^C-PIB);	Early inflammation detection;	Requires fasting for ^18^F-FDG PET	
	Sympathetic innervation (^11^C-HED);	Absolute MBF and MFR;		
	Fibrosis (^68^Ga-FAPI)	Molecular specificity		

HFpEF, heart failure with preserved ejection fraction; CMR, cardiac magnetic resonance; SPECT, single photon emission computed tomography; PET, positron emission tomography; CMD, coronary microvascular dysfunction; CAD, coronary artery disease; ATTR-CA, transthyretin cardiac amyloidosis; ^99m^Tc-PYP/DPD/HMDP, ^99m^Tc-pyrophosphate/^99m^Tc-3,3-diphosphono-1,2-propanediacetic acid/^99m^Tc-hydroxymethylene diphosphonate; ^123^I-MIBG, ^123^I-metaiodobenzylguanidine; ^99m^Tc-HMPAO, ^99m^Tc hexamethylpropyleneamine oxime; MBF, myocardial blood flow; MFR, myocardial flow reserve; ^11^C-PIB, ^11^C-Pittsburgh compound-B; ^18^F-FDG, ^18^F-fluorodeoxyglucose; MIBI, methoxy isobutyl isonitrile; LGE, late gadolinium enhancement; ECV, extracellular volume; ^68^Ga-FAPI, ^68^Gallium fibroblast activation protein inhibitor; H/M, heart-to-mediastinum; WR, washout rate; ^11^C-HED, ^11^C-hydroxyephedrine; ^68^Ga-DOTATATE, ^68^Gallium-labeled DOTA-(Tyr3)-Octreotate.

### 2.2 Identification of Specific Etiologies

#### 2.2.1 Cardiac Amyloidosis

Cardiac amyloidosis (CA) is a form of infiltrative cardiomyopathy caused by the deposition of misfolded amyloid proteins in the myocardium. The two predominant subtypes are light-chain cardiac amyloidosis (AL-CA) and ATTR-CA [17]. Recent studies have revealed that approximately 13% of patients diagnosed with HFpEF may actually have undiagnosed CA [6]. Given the significant differences in treatment strategies for AL-CA and ATTR-CA, early and accurate differentiation between these subtypes is of paramount clinical importance [18]. Although myocardial biopsy with histopathological examination is the gold standard for diagnosing CA, its invasiveness and procedural risks limit widespread clinical use. Echocardiography may detect characteristic changes such as increased ventricular wall thickness, but these findings often present only in the advanced stages of the disease [19]. CMR can demonstrate characteristic late gadolinium enhancement suggestive of amyloid deposition, yet lacks specificity for distinguishing CA subtypes. In contrast, radionuclide myocardial imaging provides a non-invasive and highly specific alternative for diagnosis and subtype differentiation. They have been recommended by international guidelines as a first-line diagnostic tool [20, 21].

Several bone scintigraphy tracers [22], such as ^99m^Tc-PYP, ^99m^Tc-3,3-diphosphono-1,2-propanediacetic acid (^99m^Tc-DPD), and ^99m^Tc-hydroxymethylene diphosphonate (^99m^Tc-HMDP), selectively bind to microcalcification proteins associated with ATTR-CA and exhibit high diagnostic specificity. They have been widely used for non-invasive screening and diagnosis of ATTR-CA. Diagnostic criteria include myocardial radiotracer uptake ≥ grade 2 or heart-to-contralateral lung ratio (H/CL) ≥1.5 (≥1.3 for 3-hour imaging) [23, 24], which yield a sensitivity of 91–97% and specificity of 87–100% [24, 25]. When combined with the negative monoclonal immunoglobulin serum/urine test, it can exclude AL-CA (about 25% of AL patients may show grade 1 uptake), and the specificity and positive predictive value for ATTR-CA approach 100% [25].

The 2023 European Society of Cardiology (ESC) Guidelines on Cardiomyopathies [21] state that DPD/PYP/HMDP SPECT myocardial imaging is the gold standard for diagnosing ATTR-CA and it may obviate the need for myocardial biopsy. Additionally, novel amyloid-targeted PET tracers such as ^11^C-Pittsburgh compound-B (^11^C-PIB), ^18^F-florbetapir, ^18^F-flutemetamol, and ^18^F-florbetaben, specifically bind to the β-sheet structure of amyloid proteins and are suitable for detecting both AL and ATTR subtypes [26, 27]. Among these, ^18^F-florbetaben and ^11^C-PIB have shown higher affinity for AL fibrils, offering the potential for subtype differentiation. Preliminary study suggests that ^18^F-NaF PET/magnetic resonance imaging (PET/MRI) can help distinguish ATTR-CA from AL-CA, with higher myocardial standardized uptake values (SUVmax) observed in the ATTR-CA group, which strongly correlate with the biopsy-confirmed amyloid burden [28]. Quantitative SPECT analysis has demonstrated a strong correlation between PYP uptake and amyloid burden measured by CMR (r = 0.873) and was significantly associated with the severity of ATTR-CA and adverse prognosis [29]. Myocardial uptake of ^11^C-PIB also correlates closely with histologically confirmed amyloid deposition in AL-CA and independently predicts prognosis [30], outperforming traditional biomarkers such as troponin I, NT-proBNP, and free light chains [31]. In another study involving 1422 ATTR-CA patients, diffuse right ventricular radiotracer uptake was significantly associated with increased all-cause mortality (78% vs. 22%) [32]. Semi-quantitative indices such as SUV not only reflect disease severity and mortality risk but can also serve as dynamic markers for therapeutic response. For instance, during treatment with tafamidis or diflunisal, radionuclide imaging can be used to monitor changes in myocardial amyloid burden and evaluate treatment efficacy [33].

Radionuclide myocardial imaging has become a key tool for the diagnosis, classification and disease monitoring of CA. Among them, SPECT bone imaging tracers have become the first-line diagnostic tool for ATTR-CA, while PET provides more possibilities for the precise differentiation of AL-CA and ATTR-CA. In the future, with the development of new PET tracers and the advancement of quantitative analysis techniques, radionuclide myocardial imaging is expected to further enhance the early diagnosis of coronary artery disease (CAD), facilitate pathological classification, and play a more significant role in individualized treatment and prognosis assessment.

#### 2.2.2 Cardiac Sarcoidosis

Cardiac sarcoidosis (CS) is an infiltrative cardiomyopathy characterized by non-caseating granulomatous inflammation of the myocardium. If left undiagnosed or untreated, it may progress to irreversible myocardial fibrosis, leading to life-threatening arrhythmias, treatment-resistant heart failure, or sudden cardiac death. Early initiation of immunosuppressive therapy during the active inflammatory phase is crucial. In patients with poor responses to immunosuppressive and heart failure medications, implantation of a left ventricular assist device (LVAD) or heart transplantation should be considered. Therefore, early and accurate diagnosis of CS and assessment of disease activity are of great significance for timely and precise treatment and reduction of adverse cardiovascular events.

Multiple expert statements recommend ^18^F-FDG PET imaging to confirm the diagnosis in patients with suspected CS [34, 35]. The clinical utility of ^18^F-FDG PET includes [36]: (1) Early diagnosis: ^18^F-FDG PET can detect myocardial inflammation before the structural or functional changes, offering an earlier diagnostic window compared to CMR. A meta-analysis reported a sensitivity of 84% and specificity of 83% for ^18^F-FDG PET in diagnosing CS [37]. Whole-body PET imaging is also useful for identifying extracardiac sarcoidosis [38], with a sensitivity of 83% and specificity of 100% when extracardiac uptake is included. (2) Assessment of disease activity and staging: Positive ^18^F-FDG uptake reflects active inflammation. When combined with resting perfusion imaging, PET can distinguish disease stages: normal perfusion with increased ^18^F-FDG uptake suggests early inflammation; reduced perfusion with elevated uptake implies both necrosis and inflammation; matched defects in perfusion and FDG uptake imply myocardial scarring. (3) Therapy guidance and monitoring: If ^18^F-FDG PET is positive, immunosuppressive therapy is required, and ^18^F-FDG PET scans can be repeated at 3, 6 and 12 months after treatment to evaluate the therapeutic effect and guide further treatment plans. After immunosuppressive therapy, if myocardial ^18^F-FDG imaging remains positive, it is one of the indications for pacemaker implantation. (4) Prognostication: ^18^F-FDG positivity correlates with major adverse cardiovascular events (MACE) in CS.

PET/MRI holds great promise in the comprehensive assessment of CS. ^18^F-FDG PET detects early inflammatory infiltration, while MRI can precisely evaluate multiple parameters such as myocardial edema and myocardial fibrosis. The two complement each other and accurately reflect the pathological changes of CS. One case study shows that a 66-year-old male patient presented with chest pain, the biopsy was confirmed as cardiac sarcoidosis. CMR revealed late gadolinium enhancement (LGE) and high T2-weighted signal in the anterior ventricular septum area. At the same time, this area showed enhanced FDG uptake, with the maximum standardized uptake value being 6.4. After 4 months of steroid treatment, the LGE and T2W signal intensities significantly decreased, and FDG uptake completely disappeared. This patient was determined to have a good therapeutic response, and no MACE occurred during the follow-up period [39]. However, ^18^F-FDG also has some limitations. Physiological myocardial uptake of ^18^F-FDG can alter image interpretation. Due to the physiological uptake of ^18^F-FDG in normal myocardium, multiple methods such as a low-carbohydrate and high-fat diet, long-term fasting, and intravenous injection of heparin before imaging [40] to reduce the uptake of ^18^F-FDG in normal myocardium can improve the diagnostic accuracy [41].

Several studies have confirmed that ^68^Ga-1,4,7,10-tetraazacyclododecane-1,4,7,10-tetraacetic-1-acid-Nal^3^-octreotide (^68^Ga-DOTANOC, a somatostatin receptor imaging agent) and 3^′^-deoxy-3^′^-^18^F-fluorothymidine (^18^F-FLT, a thymidine analogue imaging agent) have shown high accuracy in the diagnosis of CS [42, 43]. In addition, choline analogues imaging agents (such as ^11^C-choline and ^18^F-fluorocholine) and ^11^C-methionine have shown potential application in the diagnosis of extracardiac sarcoidosis [44, 45]. However, no current research has confirmed its application in the diagnosis of CS and further clinical research is still needed for verification.

#### 2.2.3 Coronary Artery Disease Including Microvascular Dysfunction

CAD has been confirmed as one of the main pathogenic factors of HFpEF [46]. In China’s largest autopsy-based study of elderly individuals, 68.2% of HFpEF patients had coexisting CAD, and 18.2% showed evidence of chronic myocardial ischemia, providing direct pathological support for the involvement of coronary microcirculation disorders in the pathogenesis of HFpEF [47]. Notably, the clinical misdiagnosis rate of CAD in HFpEF patients reached 63.3%, and the missed diagnosis rate of myocardial infarction was over 50%, highlighting the necessity of accurately assessing both coronary macrovascular and microvascular function [47]. Both epicardial coronary artery obstruction and microvascular dysfunction can lead to myocardial ischemia.

Radionuclide myocardial perfusion imaging (MPI) is currently a widely used, evidence-based, and the most reliable non-invasive imaging for evaluating myocardial blood flow. It effectively identifies the location, extent, and severity of ischemia or infarction [48], with a diagnostic sensitivity of 82–91% and specificity of 70–90% for CAD [49]. Stress-rest MPI plays an important role in diagnosis, risk stratification and prognosis assessment of CAD, and a normal stress MPI has excellent negative predictive value for MACE [50]. In addition, radionuclide fatty acid metabolism imaging (such as ^123^I-BMIPP) [51] and glucose metabolism imaging (such as ^18^F-FDG PET) [52] use the principle of “ischemic memory” to detect recent ischemic events, even after perfusion has normalized. Research [53] shows that CMD is an established independent risk factor for HFpEF and a key pathological mechanism underlying diastolic dysfunction, yet it is undetectable by conventional coronary angiography.

SPECT and PET myocardial perfusion imaging are valuable tools for evaluating CMD. A coronary flow reserve (CFR) <2.0–2.5, in the absence of epicardial stenosis, indicates CMD [54]. The major advantage of MPI lies in its ability to quantify MBF, which is essential for diagnosing ischemia or infarction in patients with non-obstructive coronary arteries, such as in myocardial infarction with non-obstructive coronary arteries (MINOCA) and ischemia with non-obstructive coronary arteries (INOCA) [55, 56, 57]. PET, owing to its superior image quality and quantitative capabilities, enables precise calculation of MBF (in mL/g/min) and MFR—the ratio of hyperemic to resting MBF—using dynamic imaging and tracer kinetic modeling [58, 59, 60]. PET-derived MFR provides a comprehensive assessment of coronary vascular function, capturing both epicardial stenosis and CMD driven by risk factors such as diabetes, dyslipidemia, hypertension, renal dysfunction, obesity, and smoking [61, 62]. In HFpEF patients without significant epicardial stenosis, radionuclide myocardial imaging facilitates CMD detection, thereby allowing for precise diagnosis and tailored treatment strategies [63]. MFR is an important indicator for evaluating coronary artery function, with reduced values associated with increased risk of MACE. Taqueti *et al*. [53] reported that CMD identified by PET (CFR ≤2.0) strongly predicts MACE. In patients without coronary artery stenosis but with impaired diastolic function, the risk of HFpEF hospitalization significantly increases. Similarly, Neglia *et al*. [64]. demonstrated that MBF independently predicts cardiac death or heart failure, irrespective of LVEF or clinical symptoms. Moreover, combined radionuclide imaging of perfusion and metabolism can identify viable myocardium with potential for functional recovery after revascularization [65]. Therefore, PET/CT-detected CMD is helpful in screening HFpEF patients who may benefit from intensified treatment, offering a robust basis for prognosis and monitoring of therapeutic interventions.

Based on the above evidence, radionuclide myocardial imaging has multiple clinical values in the management of HFpEF patients with CAD: (1) early identification of high-risk CAD patients; (2) guiding the formulation of individualized treatment strategies; (3) objectively assessing treatment effects; (4) precisely predicting disease prognosis. These advantages make it an important tool for improving the management of HFpEF patients.

As shown in Table 3 (Ref. [24, 25, 66, 67, 68, 69, 70, 71, 72, 73, 74]), the efficacy comparisons of PET, SPECT, CMR, and echocardiography in diagnosing specific causes are as follows.

**Table 3.** **Diagnostic performance of cardiac imaging modalities for key etiologies in HFpEF**.

Etiology/Imaging modality	Modality	Tracer/Technique	Sensitivity (%)	Specificity (%)
ATTR-CA	SPECT [24, 25, 66]	^99m^Tc-PYP/DPD/HMDP	91∼97%	87∼100%
	PET [67]	^18^F-florbetapir/^11^C-PIB	80∼95%	85∼98%
	Echocardiography [68]	Apical sparing strain for ATTR	97%	90%
CS	PET [69]	^18^F-FDG	81%	82%
	CMR [70]	T2, LGE	76∼100%	76∼78%
CAD	SPECT [71]	^99m^Tc-MIBI/Tetrofosmin	82∼91%	>95%
	PET [72, 73]	^13^N-ammonia/^82^Rb	90∼100%	85∼95%
CMD	CMR [74]	stress CMR perfusion	41%	83%
	PET [73]	MBF/MFR analysis	100%	75%

HFpEF, heart failure with preserved ejection fraction; ATTR-CA, transthyretin cardiac amyloidosis; CS, cardiac sarcoidosis; CAD, coronary artery disease; CMD, coronary microvascular dysfunction; SPECT, single photon emission computed tomography; PET, positron emission tomography; CMR, cardiac magnetic resonance; ^99m^Tc-PYP/DPD/HMDP, ^99m^Tc-pyrophosphate/^99m^Tc-3,3-diphosphono-1,2-propanediacetic acid/^99m^Tc-hydroxymethylene diphosphonate; ^11^C-PIB, ^11^C-Pittsburgh compound-B; ^18^F-FDG, ^18^F-fluorodeoxyglucose; ^99m^Tc-MIBI, ^99m^technetium-methoxy isobutyl isonitrile; MBF, myocardial blood flow; MFR, myocardial flow reserve; LGE, late gadolinium enhancement.

### 2.3 Molecular Imaging of Pathophysiological Processes in HFpEF

#### 2.3.1 Inflammation

Inflammatory activation is a key mechanism in the development and progression of HFpEF. Risk factors such as overweight/obesity (particularly epicardial fat accumulation), hypertension, diabetes and chronic obstructive pulmonary disease may induce systemic inflammation, promoting ventricular remodeling and diastolic dysfunction via complex signaling pathways [75]. Accurate assessment of myocardial inflammation is therefore essential for implementing targeted therapies. Currently, clinical evaluation primarily relies on circulating biomarkers such as high-sensitivity C-reactive protein (hs-CRP), erythrocyte sedimentation rate, interleukin (IL)-1β, and IL-6, which reflect systemic rather than localized myocardial inflammation. While traditional non-invasive imaging techniques, such as ultrasound, computed tomography (CT), and MRI, are useful for evaluating anatomical structures and function, they are incapable of directly imaging or quantifying inflammatory cells or their molecular markers [76]. Nuclear molecular imaging employs radiotracers to label inflammatory cells, cytokines, receptors, enzymes, inflammatory cell metabolites, adhesion molecules, and the cellular microenvironment with radiotracers [77], thereby enabling *in vivo*, non-invasive, and dynamic assessment of myocardial inflammation. This approach offers a unique tool for investigating the pathogenesis of HFpEF and guiding precision anti-inflammatory therapies.

SPECT can assess myocardial inflammation related to HFpEF by labeling white blood cells or inflammatory factors [7, 14, 78]. For example, 99mTc hexamethylpropyleneamine oxime (^99m^Tc-HMPAO) leukocyte imaging detects inflammatory cell infiltration and quantifies inflammatory burden, though its spatial resolution is suboptimal. ^67^Ga-citrate imaging is applicable for chronic inflammation assessment, but its specificity is limited. In contrast, PET enables more precise evaluation by targeting activated inflammatory cells (such as macrophages) and their surface receptors [79]. Common PET tracers include: (1) ^18^F-FDG. A classic tracer for imaging myocardial inflammation, with uptake levels closely reflecting inflammatory activity [7]. Post-acute myocardial infarction (AMI), elevated ^18^F-FDG SUVmax reflects localized inflammatory response [80], and the extent of left ventricular uptake predicts subsequent remodeling and functional deterioration [81]. However, physiological myocardial uptake and stringent dietary protocols limit its utility. (2) ^68^Ga-DOTATATE. A specific imaging tracer for M1 macrophages, ^68^Ga-DOTATATE PET allows for sensitive detection of myocardial injury and inflammation without the need for dietary preparation, as normal myocardium shows minimal uptake [8]. (3) ^68^Ga-Pentixafor. Targeting the C-X-C chemokine receptor type 4 (CXCR4), ^68^Ga-pentixafor shows increased myocardial uptake during inflammatory states and can predict adverse remodeling in HFpEF [82]. Werner *et al*. [83] reported that the infarct-to-remote myocardium SUVmax ratio of ^68^Ga-pentixafor after AMI independently predicts major adverse cardiovascular events (HR = 4.9, *p* < 0.01). (4) The translocator protein (TSPO)-targeted tracers. TSPO, abundantly expressed on activated macrophages, is a promising target. ^11^C-PK11195 PET enables detection of myocardial inflammation even in low-metabolic states, while ^18^F-GE180 can identify early post-AMI inflammation and predict adverse left ventricular remodeling at 8 weeks [84]. In addition to the above-mentioned classic probes, other novel inflammatory molecule probes have also shown potential applications in animal and early clinical studies. For instance, ^64^Cu-DOTA-ECL1i, a C-C chemokine receptor type 2 (CCR2) imaging agent, ^64^Cu-Macrin, which reflects macrophage phagocytosis, and ^11^C-methionine have all been found to be applicable for myocardial inflammation imaging [85], and are expected to be used in clinical practice in the future.

Increased epicardial adipose tissue (EAT) is associated with poor prognosis in HFpEF and is regarded as an independent cardiometabolic risk factor [86]. Nuclear molecular imaging, with its unique advantages, is expected to play a significant role in precisely evaluating the inflammatory state of EAT [87]. ^18^F-FDG PET has been used to quantify EAT inflammation and is independently associated with atrial fibrillation [87, 88]. Evidence [89] also suggests “cross-talk” between myocardial and renal inflammation post-injury, and supports a systemic immune-metabolic interplay in HFpEF pathophysiology. Whole-body PET imaging enables simultaneous assessment of multiple organs (e.g., brain, bone marrow, arteries, kidneys, liver), which is critical for mapping organ-organ interactions and systemic inflammation in HFpEF. PET/MRI fusion offers added value by combining metabolic and anatomical information. In addition, ^18^F-FDG PET imaging can detect and quantify myocardial inflammation, providing complementary information to CMR [90]. This combined PET/MRI strategy can not only be used to assess the inflammatory burden of HFpEF but also guide precise anti-inflammatory treatment (such as IL-1β inhibitors) by evaluating disease activity, progression and monitoring the response to treatment.

In summary, nuclear molecular imaging has broken through the limitations of traditional techniques, achieving dynamic visualization, quantification, and multi-target assessment of myocardial inflammation in HFpEF. This helps to screen high-risk populations with high inflammatory burden, optimize anti-inflammatory treatment strategies (such as IL-1, IL-6 inhibitors or SGLT2 inhibitors), and guide prognosis. It provides a revolutionary tool for clarifying the inflammatory mechanism, guiding targeted therapy, and improving prognosis. In the future, it is necessary to further promote the clinical transformation of new tracers and the integration of multi-modal imaging technologies to provide a “molecular microscope” for clarifying the pathogenesis of HFpEF and will further drive the diagnosis and treatment of heart failure into the era of precision medicine.

#### 2.3.2 Myocardial Fibrosis

Myocardial fibrosis is a central pathological mechanism in the occurrence and development of HFpEF [1]. It impairs ventricular compliance through dual pathways of interstitial fibrosis (excessive collagen deposition) and replacement fibrosis (scar repair), leading to progressive diastolic dysfunction and significantly increasing the risk of cardiovascular death and rehospitalization for heart failure [91]. This key pathological process makes it one of the most promising therapeutic targets for HFpEF. Early and accurate identification and intervention in the fibrotic process are necessary for reversing ventricular remodeling and improving patient prognosis. Traditional diagnostic methods have obvious limitations: although endomyocardial biopsy is the gold standard [92], it is invasive and susceptible to sampling bias; CMR using LGE can only identify focal late-stage fibrosis [93]. Serum biomarkers (e.g., procollagen type I C-terminal propeptide, PICP, procollagen type I N-terminal propeptide, PINP) are limited in clinical application due to the lack of cardiac specificity. This diagnostic dilemma is being overcome by nuclear molecular imaging. By specifically targeting key molecular events of fibrosis (such as fibroblast activation, collagen synthesis and cross-linking), it has achieved a paradigm shift from “anatomical imaging” to “visualizing pathological processes”, laying the technical foundation for the era of precision medicine in HFpEF.

Fibroblast activation is a key link in the fibrotic process, and is reflective of the early stage, activity and reversibility of fibrosis. Recently, radiolabeled molecular probe targeting FAP, represented by ^68^Ga-FAPI PET, has been able to detect the activity of myocardial fibrosis in HFpEF by binding to FAP expressed on activated fibroblasts. Uptake in HFpEF patients is approximately 2.1-fold higher than that in healthy controls (*p* < 0.01). Moreover, it can predict the risk of left ventricular remodeling after myocardial infarction (area under the ROC curve (AUC) = 0.89) and subclinical fibrosis in patients with diabetes or obesity [94, 95]. A study has demonstrated [9] that ^68^Ga-FAPI uptake is elevated in the early phases of fibrosis. Thus, ^68^Ga-FAPI PET facilitates early identification of active myocardial fibrosis, providing an important foundation for timely therapeutic intervention aimed at preventing adverse ventricular remodeling. Furthermore, although ^68^Ga-FAPI demonstrates promising potential for imaging-based assessment of myocardial fibrosis, its diagnostic specificity, particularly in the early detection phase, still requires further validation and confirmation through large-scale clinical trials.

Studies involving novel tracers such as ^99m^Tc-CBP1495 (a collagen-binding probe) and ^18^F-Alfatide II (targeting αvβ3 integrin) [96, 97] have shown that PET imaging holds promise for evaluating the severity of fibrosis and monitoring its progression. Future multi-center trials (such as the FIBRO-HFpEF study) are warranted to delineate subtype-specific regulation strategies for fibroblasts (repair-promoting vs. fibrosis-promoting), and to advance the development of new probes targeting enzymes such as lysyl oxidase (LOX) and matrix metalloproteinases (MMPs) [98]. Ultimately, a comprehensive fibrosis management framework—encompassing early diagnosis, precise phenotyping, dynamic monitoring, and prognostic evaluation—should be established to redefine the therapeutic paradigm of HFpEF.

Nuclear molecular imaging has broken through the limitations of traditional imaging, achieving non-invasive, dynamic and quantitative assessment of myocardial fibrosis in HFpEF. It plays a significant role in the precise classification, treatment monitoring, and prognosis prediction of HFpEF patients. In the future, it is necessary to further promote the clinical transformation of new tracers and conduct large-scale, multi-center studies to verify their clinical application value. PET/MRI combined strategy is expected to become a new standard for the assessment of myocardial fibrosis in HFpEF. Exploring the molecular imaging strategy of fibrosis-inflammation interaction will help to formulate and optimize individualized and precise treatment strategies.

#### 2.3.3 Sympathetic Innervation Imaging

The autonomic nervous system plays an important role in regulating cardiac function, including heart rate, myocardial contractility and blood pressure. In heart failure, particularly HFpEF, sympathetic nervous system (SNS) overactivation—as well as activation of the renin–angiotensin–aldosterone system (RAAS)—contributes to adverse ventricular remodeling, increases the risk of arrhythmias, and is strongly associated with poor prognosis [99]. Nuclear imaging of cardiac sympathetic innervation has emerged as a valuable tool in clinical research and patient management [100]. The most widely used approach for evaluating cardiac sympathetic function involves imaging presynaptic nerve terminals using radiolabeled catecholamine analogues. Among them, ^123^I-metaiodobenzylguanidine (^123^I-MIBG) is a widely used SPECT imaging, while ^11^C-hydroxyephedrine (^11^C-HED) is often employed for PET imaging [100]. These radiotracers provide direct assessment of myocardial sympathetic activity and are useful for detecting autonomic dysfunction, risk stratification, prognostication, individualized therapy, and treatment monitoring in HFpEF.

Multiple studies [10, 99] have shown that ^123^I-MIBG imaging is more valuable than traditional indicators such as LVEF and BNP in risk stratification of heart failure patients and has gradually become an important tool for assessing prognosis [101]. The heart-to-mediastinum (H/M) uptake ratio and myocardial clearance rate are independent predictors of adverse outcomes across various heart failure etiologies [101]. Several studies [102, 103, 104] have shown that the H/M ratio in patients with heart failure is closely related to their poor prognosis, regardless of whether they have HFrEF, heart failure with mid-range ejection fraction (HFmrEF), or HFpEF. The overall sympathetic nerve function of the heart and regional heterogeneity of cardiac innervation are also closely related to malignant ventricular arrhythmias. A study [100] found that if there is a region with relatively good perfusion but damaged nerves near the area of the myocardial scar, namely “neuro-perfusion mismatch”, the patient is more prone to arrhythmias. The PAREPET study found through ^11^C-HED PET inflammation imaging that the “neuro/perfusion mismatch” area is closely related to the occurrence of sudden cardiac death in patients with ischemic heart disease, and is not affected by LVEF, infarction size and BNP levels [105]. In addition, nuclear neuroimaging can assess the effects of therapeutic interventions on sympathetic activity. A study [106] found that treatment with beta-blockers, aldosterone antagonists, and continuous positive airway pressure (CPAP) ventilation, significantly improves ^123^I-MIBG or ^11^C-HED uptake, suggesting that sympathetic dysfunction is at least partially reversible. In patients with diabetes, cardiac autonomic neuropathy (CAN) is a common and serious complication. Among diabetic patients with heart failure, those with an H/M ratio <1.6 have a threefold increased risk of disease progression compared to those with H/M ratio >1.6 [102]. Both ^11^C-HED and ^123^I-MIBG imaging can detect early CAN and monitor its progression over time [106]. Notably, nerve function may partially recover with intensive glycemic control, supporting early intervention as a strategy to delay or prevent neuropathy progression and improve heart failure outcomes in diabetic patients.

Despite its clinical promise, widespread adoption of cardiac sympathetic innervation imaging faces technical challenges. ^11^C-labeled tracers like ^11^C-HED, with a half-life of only 20 minutes, must be synthesized on-site using a cyclotron, which is difficult to achieve in most medical institutions. In contrast, the ^18^F-labeled tracers with a half-life of up to 110 minutes, such as ^18^F-fluorobenzylguanidine and ^18^F-m-fluorobenzylguanidine, have greater practical potential [103] and hold promise for broader clinical implementation.

Although sympathetic innervation imaging has demonstrated strong prognostic value in heart failure and arrhythmias, prospective studies are still needed to demonstrate its direct impact on clinical decision-making. Consequently, this technique has not yet been incorporated into heart failure management guidelines [7]. Nevertheless, in selected high-risk or borderline patients—particularly those being considered for catheter ablation or device therapy—sympathetic imaging may serve as a valuable adjunctive tool for risk assessment and management.

#### 2.3.4 Metabolic Imaging

Metabolic abnormalities play a key role in the pathogenesis of HFpEF. Patients with metabolic syndrome, particularly those with obesity or diabetes, frequently exhibit myocardial energy metabolism disorders, which are increasingly recognized as key contributors to impaired diastolic function. Under stress, the heart undergoes substrate shifts as a compensatory mechanism in pathological remodeling [107]. A comprehensive understanding of this metabolic reprogramming is essential not only for elucidating HFpEF pathophysiology but also for informing the development of targeted therapeutic strategies. Metabolic alterations in HFpEF are characterized by disruptions across multiple pathways, including fatty acid oxidation, glucose oxidation, glycolysis, ketone body metabolism, and branched-chain amino acid (BCAA) metabolism [108]. Nuclear molecular imaging provides a powerful tool for a comprehensive assessment of these metabolic alterations. Currently, ^18^F-FDG PET/CT is widely used in clinical practice to non-invasively evaluate the myocardial glucose metabolic status, especially for HFpEF patients with concomitant disorders of glucose and lipid metabolism. It can reveal changes in myocardial glucose uptake, a metabolic feature that may be closely related to diastolic dysfunction. However, ^18^F-FDG imaging is subject to several limitations: its uptake is influenced by insulin sensitivity, dietary status and cardiac load, resulting in poor reproducibility of the results. More importantly, it only reflects a single link of glucose metabolism and is difficult to comprehensively evaluate the myocardial metabolic network. Therefore, it is still necessary to develop more targeted and stable tracers to achieve a more accurate quantitative assessment of myocardial metabolic status.

Beyond glucose metabolism, the myocardium relies heavily on fatty acids for energy. To more comprehensively assess the myocardial metabolic status, researchers have also employed imaging agents such as ^18^F-fluoro-6-thia-heptadecanoic acid (^18^F-FTHA) and ^11^C-palmitate [109] to detect myocardial fatty acid uptake, thereby analyzing the balance of myocardial substrate metabolism. Some researchers [110] used ^18^F-FTHA and ^18^F-FDG as tracers and found through PET imaging that compared with normal individuals, the rate of free fatty acid uptake in the myocardium of heart failure patients decreased, while the rate of glucose uptake increased. PET/CT can assess the therapeutic effect of drugs on heart failure by comparing the myocardial metabolic changes before and after drug treatment in heart failure patients. ^11^C-acetate (^11^C-ACE) myocardial PET imaging can evaluate the aerobic metabolism of the myocardium in heart failure patients [111]. In patients with heart failure, aerobic metabolism in the myocardium increases, and the clearance rate of ^11^C-ACE is significantly lower than that of normal individuals. After treatment with beta-blockers, aerobic metabolism decreases and the clearance rate of ^11^C-ACE increases. In addition, myocardial peripheral efficiency has also become one of the indicators of concern in recent years. It can be evaluated by combining ^11^C-ACE PET to measure myocardial oxygen uptake and the left ventricular peripheral work estimated by the product of stroke volume and mean arterial pressure [111]. Studies [112] suggest that the efficiency of damaged myocardium is even more effective than the ejection fraction in predicting the poor prognosis of patients.

In summary, radionuclide myocardial metabolic imaging provides critical insights into the energy metabolism of the HFpEF myocardium. It shows promises for early disease detection, the response and evaluation of treatments, and precision therapy. Future research should focus on developing a multimodal metabolic assessment system to more accurately guide clinical decision-making.

### 2.4 Cardiac Function Evaluation

Gated myocardial perfusion imaging (GMPI) is a widely used nuclear cardiology technique in clinical practice. By using the R wave of the electrocardiogram as a trigger, GMPI segments each cardiac cycle into 8 or 16 equal phases, allowing sequential acquisition of myocardial images from end-systole to end-diastole [113]. GMPI can accurately obtain the left ventricular volume-time curve, and further calculate diastolic function parameters including peak filling rate (PFR) and time to peak filling (TPF), as well as indices of left ventricular mechanical dyssynchrony (LVMD), such as diastolic phase bandwidth, phase standard deviation (PSD), and phase entropy [113]. These objective metrics allow comprehensive assessment of both diastolic function and intraventricular synchrony. Study has demonstrated a strong correlation between diastolic PSD and phase bandwidth derived from GMPI phase analysis and the degree of diastolic dyssynchrony measured by tissue Doppler imaging (TDI) [114] GMPI-derived indices exhibit superior reproducibility, making them well-suited for longitudinal monitoring. In HFpEF, patients frequently exhibit significant diastolic dyssynchrony despite preserved systolic synchrony, a phenomenon linked to increased myocardial stiffness due to fibrosis and disrupted energy metabolism [115, 116, 117]. Diastolic dyssynchrony represents not only a hallmark pathological feature of HFpEF but also an independent predictor of adverse outcomes [118]. It correlates with worsening cardiac structure and function and can guide risk stratification. For instance, post-myocardial infarction patients with a diastolic PSD >55.5° have a significantly increased risk of MACE [119]. Cardiac resynchronization therapy (CRT) for asynchrony-related events has been proven to reduce the morbidity and mortality of heart failure patients [120]. Given this evidence, the timely and accurate diagnosis of LVMD in patients with heart failure is of crucial clinical significance. Furthermore, right ventricular (RV) function also plays a crucial role in HFpEF [121, 122]. Radionuclide ventriculography can simultaneously obtain the right ventricular volume and functional parameters. When the RV is difficult to be clearly displayed by ultrasound, it provides more accurate and repeatable data, which is helpful for a comprehensive assessment of biventricular function [123].

In conclusion, GMPI-derived functional parameters serve as non-invasive, reproducible imaging biomarkers for diastolic function assessment in HFpEF. These may complement clinical decision-making in risk stratification and treatment monitoring. Future prospective studies are warranted to validate their role in guiding personalized management strategies.

### 2.5 Future Directions and Perspectives

Radionuclide myocardial imaging has shown considerable clinical value in the diagnosis and management of HFpEF, particularly in identifying specific etiologies, uncovering key pathophysiological mechanisms, and enabling individualized risk stratification. However, its broader clinical adoption remains constrained by several challenges. High costs, limited availability of PET imaging systems, and the technical complexity of advanced tracers have restricted their widespread use, especially in resource-limited settings. In contrast, SPECT imaging using ^99m^Tc-labeled tracers is more accessible and cost-effective, making it a practical first-line modality. A tiered diagnostic strategy—employing SPECT for initial screening (e.g., ^99m^Tc-PYP for ATTR-CA) and reserving PET for advanced assessment in tertiary centers—is therefore recommended. Additionally, the integration of artificial intelligence (AI)—based quantitative tools may enhance diagnostic reproducibility and reduce inter-observer variability. Despite technological progress, many radionuclide imaging parameters still lack standardized diagnostic thresholds and validation in large-scale, multicenter studies. Furthermore, several promising radiotracers, such as FAPI and FLT, remain in the early stages of clinical development, necessitating further research to confirm their utility in early detection, disease monitoring, and therapeutic guidance.

Future studies should focus on several key areas. First, the integration of multimodal imaging techniques (PET/CT, PET/MR) with AI-driven image analysis will enable high-throughput, multi-dimensional assessment of HFpEF mechanisms. Second, developing more specific molecular probes targeting inflammation, fibrosis, and metabolic dysfunction may support a shift from phenotype-based to mechanism-based disease classification. Third, building comprehensive clinical models that combine nuclear imaging biomarkers with conventional tools—such as NT-proBNP, CMR, and functional scoring systems—can refine therapeutic decision-making. Fourth, evaluating the role of nuclear imaging in predicting treatment response will be crucial, particularly with the rise of SGLT2 inhibitors, anti-inflammatory agents, and anti-fibrotic therapies. Finally, advances in AI are expected to streamline image processing, optimize radiation dosing, and facilitate individualized prediction of prognosis through multiparametric modeling. In addition, enhancing patient education is of great significance in improving their acceptance and compliance with the process of radionuclide imaging. Adequate informed communication about the benefits and limitations of the examination can help increase patient compliance, especially for elderly patients or those with multiple underlying diseases.

## 3. Conclusion and Future Perspectives

HFpEF, often regarded as the “final frontier” in the management of heart failure, urgently demands innovative technologies to overcome persistent diagnostic and therapeutic challenges. Nuclear myocardial imaging, by virtue of its unique strengths in molecular imaging, is currently spearheading the paradigmatic transformation of the diagnosis and treatment mode of HFpEF. This technique not only transcends the constraints of traditional imaging, achieving a cognitive leap from macroscopic phenotypes to microscopic mechanisms, but also, via a multi-parameter and quantitative assessment system, furnishes crucial technical support for the precise medical practice of HFpEF. Current evidence strongly supports that nuclear imaging possesses three core values in the diagnosis and treatment of HFpEF: at the diagnostic level, it can precisely identify special etiologies and key pathological mechanisms; at the therapeutic level, it is capable of objectively assessing therapeutic effects and predicting treatment responses; at the management level, it can realize individualized prognosis assessment and the entire process management.

Looking forward to the future, with the rapid development of molecular imaging technology and the cross-integration of multiple disciplines, radionuclide myocardial imaging will realize three major transitions in the field of HFpEF: from an auxiliary diagnostic tool to a decision support system, from single-parameter evaluation to multi-omics integration, and from preclinical research to clinical translation. These transitions will profoundly affect the diagnosis and treatment strategies of HFpEF, ultimately achieving a qualitative leap from “symptomatic treatment” to “etiological treatment” and from “group-based regimens” to “individualized regimens”, providing crucial technical support for enhancing the prevention and treatment of heart failure.

## Abbreviations

SPECT, single photon emission computed tomography; PET, positron emission tomography; MRI, magnetic resonance imaging; MPI, myocardial perfusion imaging; MFR, myocardial flow reserve; MBFR, blood flow reserve; MBF, myocardial blood flow; MACE, major adverse cardiovascular events; LVEF, left ventricular ejection fraction; LVAD, left ventricular assist device; HFrEF, heart failure with reduced ejection fraction; HFpEF, heart failure with preserved ejection fraction; GMPI, Gated myocardial perfusion imaging; FAP, fibroblast activation protein; EAT, excessive epicardial adipose tissue; CXCR4, C-X-C chemokine receptor type 4; CS, cardiac sarcoidosis; CMR, cardiac magnetic resonance; CMD, coronary microvascular dysfunction; CFR, coronary flow reserve; CCR2, C-C chemokine receptor type 2; CAD, coronary artery disease; CA, cardiac amyloidosis; ATTR-CA, transthyretin cardiac amyloidosis; AMI, acute myocardial infarction; AL-CA, light chain cardiac amyloidosis; ^99m^Tc-PYP, ^99m^Tc-pyrophosphate; ^99m^Tc-MIBI, ^99m^technetium-methoxy isobutyl isonitrile; ^99m^Tc-HMDP, ^99m^Tc-hydroxymethylene diphosphonate; ^99m^Tc-DPD, ^99m^Tc-3,3-diphosphono-1,2-propanediacetic acid; ^68^Ga-DOTATATE, ^68^Gallium-labeled DOTA-(Tyr3)-Octreotate; ^18^F-FDG, ^18^F-fluorodeoxyglucose; ^123^I-MIBG, ^123^I-metaiodobenzylguanidine; ^123^I-BMIPP, Iodine-123 β-methyl iodophenyl pentadecanoic acid; ^11^C-PIB, ^11^C-Pittsburgh compound-B; ^11^C-HED, ^11^C-hydroxyephedrine.
